# Book review: The fractal geometry of the brain

**DOI:** 10.3389/fnins.2022.1078376

**Published:** 2022-11-23

**Authors:** Karolina Armonaite, Livio Conti, Franca Tecchio

**Affiliations:** ^1^Faculty of Psychology, Uninettuno University, Rome, Italy; ^2^Laboratory of Electrophysiology for Translational NeuroScience, Institute of Cognitive Sciences and Technologies—Consiglio Nazionale delle Ricerche, Rome, Italy; ^3^Faculty of Engineering, Uninettuno University, Rome, Italy; ^4^INFN—Istituto Nazionale di Fisica Nucleare, Sezione Roma Tor Vergata, Rome, Italy

**Keywords:** fractal dimension, neurodynamics, nervous system, complexity, multiple scales

## Introduction

At a crucial turning point, we can now witness the transition from the promotion of computational methods in neuroscience to a change of habits in several frontiers of neurosurgery, neuroprosthetics, neuromodulation, and basic neuroscience. In our laboratory's experience, from the indications that the same triadic principle governs the functioning of neuronal networks when the node of the network is a single neuron, a group of neurons, or large neuronal areas, we have come to the view that the expression of neuronal network activity has fractal properties.

Fractality is a concept introduced by mathematician Benoit Mandelbrot, one of the first to note that natural phenomena cannot be described by traditional Euclidean geometry, as lightning, clouds, and mountains are not perfect lines, rectangles, or cones, but have a more sophisticated structure. In particular, structures such as trees present similar patterns to themselves at different scales. A non-integer topological dimension characterizes these systems and defines their name, fractals. The brain is a complex system with various structures that exhibit a fractal property. It is interesting to note that, probably as an effect of similar governing principles presenting themselves at different scales of the neuronal network, electrical activity with a time course presenting fractal properties arises. Here, Antonio di Ieva's “The Fractal Geometry of the Brain,” 2016 book is a unique opportunity for neuroscientists and clinicians to take a new, broader view of understanding the brain in scientific and clinical settings *via* fractal properties (Di Ieva, 2016).

## Summary of the book

Thanks to an increasingly clear view of the nervous system as a neuronal network expressing the properties of a complex system; concepts such as non-linear dynamics or fractality of the brain structure and processes are more frequently encountered in neuroscience. The book is a clear example of this approach, so chapter by chapter we are introduced to a novel way of thinking about the brain.

The first part of the book gives a well-integrated overview of fractal geometry and its neuroscientific applications. In the first chapter, Di Ieva himself introduces the birth of fractal analysis in biomedicine and the main concepts for clinical applications. Further chapters lay out fundamental principles of fractality in general, and with not heavy but mathematical rigor introduce the features of fractal objects and the methods for estimating their fractal dimension D_F_. In the second part, the morphology of the brain tissues, nervous system, and cranial structures is described in the framework of fractal geometry. These chapters provide quantitative tools to investigate the nervous system components, such as dendrites, microglia, and synapses as fractals, because the fractal analysis has the potential to classify the intricate structures of anatomical and functional complexity. In the third part of the book, great attention is given also to the fractals in clinical neuroscience. Many authors lay down the concepts of spatial and temporal neurodynamic fractal analysis in predicting neurodegenerative diseases and attempting to determine the fractal properties of neurological disorders and physio-pathologies. Finally, the book is concluded with the fourth part, which is dedicated to computational fractal-based neurosciences. These chapters cover the possible applications of fractal complex patterns simulations and modeling healthy and impaired brain dynamics and connectivity as well as draw future connections with artificial intelligence.

## Evaluation of the book's content

The book elegantly summarizes and gradually unveils the fractal geometry approach to investigate the brain, from principle to clinical application and computational analysis, and finally sketches future perspectives toward fractal electronics and computational intelligence.

Initially, Antonio di Ieva expresses a few critics, citing Benoit Mandelbrot, that fractals are not everywhere and are not a panacea. We also believe that fractal geometry has to be applied with a little heed. Though, as the Author notices, fractal geometry is a unique way to deal with systems that cannot be studied with only traditional Euclidean geometry, as the brain is. Therefore, agreeing with the Author, we suggest to look at the brain as a fractal object with respect to its structure and function.

Even though in most cases real-world fractals possess statistical self-similarity, the measures cited in the book are suitable with many limits for empirical application. Despite that, most of those limits are introduced or partially discussed in the chapters.

Nevertheless, we agree to think of the brain as a self-similar structure with multiple scales that, mediated by individual probabilistic events, shapes the neurodynamics of neuronal pools. However, we would emphasize the importance of the careful selection of appropriate fractal measures, as evidence suggests that they may determine sensitivity to physiological phenomena (Kesić and Spasić, 2016).

On the basis of the well-described analyses given in the book, from a morphological point of view, the nervous system can have fractal properties from a cell to the whole network, as well as the neuronal ongoing activity might possess fractal behavior. On the other side, one might consider the necessity of a rigorous demonstration of the stability of internal structures and invariance in time and across subjects.

## Discussion of the book's content in light of the current needs of the community

The idea of finding new methods to evaluate the properties of the brain in research and clinical settings is to complement the existing methods and allow to facilitate occurring difficulties as well as to draw future perspectives in the automation of real-time data analysis. This book gives a priceless overview of how to deploy these new procedures.

The repeatability at different scales yields existing patterns of neurodynamics, hemodynamics, or cerebrovascular system and changes in them suggest pathological alterations, in consequence, helps in recognizing aneurysms, tumors, and demyelinating processes. Fractal features of electrophysiology appeared to be significant in epilepsy, depression, and schizophrenia diagnosis too (Sharma et al., 2017; Lebiecka et al., 2018). Furthermore, the aging brain could be recognized by its structural and functional complexity changes and in fact, it has already been demonstrated that fractal dimension can be one of the biomarkers in diagnosing Alzheimer's disease (Smits et al., 2016; Al-Nuaimi et al., 2017). Therefore, an estimation of fractal measure could be a non-invasive tool to detect several pathologies.

In addition, the book suggests the fractal analysis of EEG signals to be useful for sleep staging and depth of anesthesia evaluation. This method is practical because it does not require the removal of artifacts and can be applied to raw data. Indeed, a secondary result of possible sleep staging with fractal dimension estimate was found by Armonaite et al. (2022), where different sleep stages were distinguished from 1-min intracranial EEG recordings.

In general, fractal-based methods provided in this book for brain structure and function assessment are a huge step further toward computational neuroscience for understanding the brain, more efficient disease detection, and even neurological treatment by applying bioelectronics.

## Author contributions

KA wrote the manuscript supervised by FT and LC who provided suggestions and edits for revision. All authors approved the submitted version.

## Funding

The work received financial support from POR FESR Lazio Innova 2014–2020, Prog. 28109, Digital Helpers per e-Communities in Sanità [DHelp4H], prot. Gecoweb A0320-2019-28109.

## Conflict of interest

The authors declare that the research was conducted in the absence of any commercial or financial relationships that could be construed as a potential conflict of interest.

## Publisher's note

All claims expressed in this article are solely those of the authors and do not necessarily represent those of their affiliated organizations, or those of the publisher, the editors and the reviewers. Any product that may be evaluated in this article, or claim that may be made by its manufacturer, is not guaranteed or endorsed by the publisher.
